# Interim PET/CT based on visual and semiquantitative analysis predicts survival in patients with diffuse large B‐cell lymphoma

**DOI:** 10.1002/cam4.2404

**Published:** 2019-07-10

**Authors:** Xiaoqian Li, Xun Sun, Juan Li, Zijian Liu, Mi Mi, Fang Zhu, Gang Wu, Xiaoli Lan, Liling Zhang

**Affiliations:** ^1^ Cancer Center Union Hospital, Tongji Medical College, Huazhong University of Science and Technology Wuhan China; ^2^ Department of Nuclear Medicine Union Hospital, Tongji Medical College, Huazhong University of Science and Technology Wuhan China

**Keywords:** deauville 5‐point scale, diffuse large B‐cell lymphoma, interim PET/CT, SUVmax reduction, tumor burden

## Abstract

**Purpose:**

The role of interim ^18^F‐FDG PET/CT (iPET/CT) in diffuse large B‐cell lymphoma (DLBCL) remains controversial. The purpose of this study was to assess the prognostic value of iPET/CT in patients with newly diagnosed DLBCL according to visual and semiquantitative interpretation methods.

**Methods:**

A total of 129 newly diagnosed DLBCL patients with baseline PET/CT data were retrospectively screened. The iPET/CT findings were evaluated by the Deauville 5‐point scale (DS) and ΔSUVmax. Furthermore, the reduction in SUVmax incorporated with tumor size (ΔSUVmax*ΔSLD) was calculated. The optimal cutoff values of ΔSUVmax and ΔSUVmax*ΔSLD were determined by receiver operating characteristic (ROC) analysis. Kaplan‐Meier analysis was applied to test for the influence of prognostic values. Univariate and multivariate analyses were conducted to examine the potential independent impacts of iPET/CT.

**Results:**

Seventy‐seven patients with PET/CT images acquired both at baseline and after four cycles of chemotherapy were finally enrolled. The optimal cutoff values for ΔSUVmax and ΔSUVmax*ΔSLD were 74% and 30%, respectively. After a median follow‐up of 23 months, iPET/CT findings were significant predictors of PFS and OS whenever iPET/CT was interpreted by DS, ΔSUVmax, or ΔSUVmax*ΔSLD methods. ΔSUVmax‐based methods were more accurate than those based on DS. The IPI, DS, ΔSUVmax, and ΔSUVmax*ΔSLD were predictive in univariate analyses. However, in the multivariate analysis, only IPI and ΔSUVmax remained independent predictors of PFS and OS.

**Conclusions:**

Interim PET/CT may help to identify DLBCL patients with different prognoses. ΔSUVmax analysis shows the best accuracy and the strongest predictive value among these three methods. ΔSUVmax*ΔSLD may be a promising parameter to interpret iPET/CT images, reflecting both the changes in tumor size and metabolic activity.

## 1 INTRODUCTION

Diffuse large B‐cell lymphoma (DLBCL) is the most common subtype of non‐Hodgkin lymphoma.^1^ Although the addition of the anti‐CD20 monoclonal antibody rituximab to CHOP has dramatically improved treatment outcomes in DLBCL patients, approximately 30%‐40% of patients will relapse after first‐line treatment.^2^ Therefore, defining the prognosis for individual patients as early as possible is very important.

^18^F‐fluorodeoxyglucose positron emission tomography/computed tomography (^18^F‐FDG PET/CT) imaging is currently widely used in the management of DLBCL. PET/CT imaging provides both anatomical and functional information that enables it to fundamentally alter staging, guiding the choice of treatment modality, as well as monitoring and assessing treatment response of lymphomas.^3^ The 2014 guidelines recommend performing baseline and end‐of‐treatment PET/CT in clinical practice for patients with DLBCL; however, the purpose and contribution of interim PET/CT (iPET/CT) remain controversial^4^ because studies have shown mixed results.

The debate of iPET/CT may be partly attributed to the lack of uniform operating time and evaluation methodologies. Varied timing of iPET/CT has been reported in the literature, and in most studies, iPET/CT was performed after two or four cycles of chemotherapy.^5^, ^6^, ^7^ Tumor glucose metabolism in the early stage of disease has been recognized as a favorable prognostic factor in DLBCL regardless of the times at which iPET is performed.^8^

Determining the best method to interpret iPET is also a significant challenge. Although Deauville 5‐point scale (DS) was recommended as a standard method, the variable positive predictive value (PPV) of iPET using visual analysis is a main point of contention. According to DS assessment, the PPV of iPET ranges from 33% to 100%.^9^, ^10^, ^11^ Semiquantitative methods, such as standardized uptake value reduction (ΔSUVmax) and metabolic tumor volume reduction (ΔMTV), have been explored to improve the accuracy of iPET. Several studies have demonstrated that patients who achieved a ΔSUVmax beyond the cutoff value on an iPET scan had favorable outcomes.^12^, ^13^, ^14^ Increasing evidence supports that ΔSUVmax seems better than DS when interpreting iPET results in DLBCL patients.^6^, ^14^, ^15^, ^16^, ^17^ However, iPET analysis needs further validation in clinical trials. Neither the DS nor the ΔSUVmax method considers the tumor burden during treatment. ΔMTV reflects changes in tumor metabolic activity as well as tumor volume, but it cannot be easily obtained from routine practice PET scans because special software is needed.^18^

Whenever DS, ΔSUVmax, or ΔMTV is used to interpret iPET/CT, only the PET data are evaluated, and the CT data are ignored. As in the Lugano response criteria, the response is assessed according to PET results using DS interpretation. Changes in tumor size on CT scan seem unimportant in FDG‐avid lymphomas, such as DLBCL. In fact, a combination of changes in tumor size on CT and changes in SUVmax has been explored and may predict progression‐free survival (PFS) better than either test alone in Hodgkin's lymphoma.^19^, ^20^ Furthermore, the Lugano criteria were challenged more recently.^21^, ^22^ The change in tumor burden became a very important element in the International Working Group consensus response evaluation criteria in lymphoma (RECIL 2017).^21^ In the RECIL 2017 criteria, partial response (PR) is defined as a reduction in the sum of longest diameters (SLD) of targets by ≥30% but with a positive PET (DS 4‐5). Furthermore, stable disease (SD) and progressive disease (PD) are defined based on changes in SLD, irrespective of PET scan results. Therefore, SLD reduction (ΔSLD) on CT scans may still be a very important surrogate for tumor burden, which is also recommended by response evaluation criteria in solid tumors (RECIST 1.1),^23^ even though PET scan is recommended as a standard care for DLBCL response evaluation. In the present study, we investigated the role of ΔSLD combined with ΔSUVmax (ΔSUVmax*ΔSLD) in DLBCL.

Herein, we retrospectively evaluated 129 newly diagnosed DLBCL patients, and visual and semiquantitative methods were used to assess iPET/CT data. Changes in metabolic activity (DS and ΔSUVmax) on PET scans and tumor burden alternations (ΔSLD) on CT scans were measured. The aim of this study was to investigate the prognostic values of different iPET/CT methods, including DS, ΔSUVmax, and ΔSUVmax*ΔSLD, in DLBCL patients.

## 2 MATERIALS AND METHODS

### 2.1 Patients and treatment

We retrospectively screened 129 newly diagnosed DLBCL patients with baseline PET/CT at Cancer Center, Union Hospital, Tongji Medical College, Huazhong University of Science and Technology, Wuhan, between October 2013 and January 2017. The inclusion criteria were as follows: patients with histologically proven DLBCL only, patients who received interim PET/CT after two or four cycles of chemotherapy, and patients treated with a standard‐dose CHOP (cyclophosphamide, doxorubicin, vincristine, and prednisone) or R‐CHOP (rituximab plus CHOP) in cycles of 21 days each. The study was approved by the ethics committee of the college, and a waiver of consent was allowed by the ethics committee because there were no conflicts of interest or damages to patients, and patient data confidentiality was guaranteed according to the requirements of the ethics committee.

Seventy‐seven patients were finally enrolled, and the clinical characteristics, treatment results, and follow‐up were obtained from a review of medical records.

### 2.2 PET/CT imaging

PET/CT scans were performed 2 weeks after the second or the fourth cycles of chemotherapy. Baseline and interim PET/CT were performed on the same scanner (Discovery VCT, GE Healthcare, Milwaukee, WI). After at least 6 hours of fasting with an optimal blood glucose level lower than 8 mmol/L, patients were injected intravenously with 5 MBq/kg of ^18^F‐FDG. Images were acquired from the skull base to the upper thighs 60 minutes after the injection. Images were then corrected for attenuation with low‐dose CT data, reconstructed with a three‐dimensional iterative algorithm, and finally fused with CT images.

### 2.3 Visual analysis of PET/CT

PET/CT images were reviewed by two certified nuclear medicine physicians (Sun X and Lan X) and two oncologists (Li X and Zhang L). The iPET/CT data were visually interpreted according to DS: score 1, no FDG uptake above background; score 2, FDG uptake ≤ mediastinum uptake; score 3, FDG uptake > mediastinum uptake but ≤ liver uptake; score 4, FDG uptake moderately > liver uptake; and score 5, FDG uptake markedly higher than liver uptake and/or new lesions were present.

### 2.4 Semiquantitative analysis of PET/CT

PET/CT images before and during treatment were displayed using a fixed SUV scale and color table. ΔSUVmax calculation was performed as described elsewhere.^14^ Briefly, for each PET scan, the tumor with the most intense FDG uptake was identified using a maximum intensity projection display with a graded color scale with yellow indicating the SUVmax. To calculate ΔSUVmax, if a residual lesion was present, the SUVmax of iPET was measured on the hottest focus of the residual lesion. If no lesions were identified on iPET, SUVmax was drawn in the same area on iPET as on PET0. The percentage reduction in SUVmax (ΔSUVmax) between PET0 and iPET was calculated as follows: ΔSUVmax = (PET0 SUVmax‐iPET SUVmax)/PET0 SUVmax × 100%.

In addition, SLD was calculated according to response evaluation criteria in solid tumors (RECIST 1.1) and RECIL 2017. In general, the largest most reproducible lesions are selected to follow as target lesions, and the same lesion chosen to make the SUVmax calculation must be included. On CT scans, if a residual lesion was present, actual measurements, even for target lesions＜10 mm, were recorded. If target lesions actually disappeared, a default measurement of 0 mm was recorded. The percentage reduction in SLD (ΔSLD) between baseline CT (CT0) and interim CT (iCT) was calculated: ΔSLD = (CT0 SLD‐iCT SLD)/CT0 SLD × 100%. Then, the product of ΔSLD and ΔSUVmax (ΔSUVmax*ΔSLD) was calculated as the greatest diameter (mm) of the same lesion chosen to make SUVmax calculation.

### 2.5 Clinical outcomes

Progression‐free survival and overall survival (OS) were clinical end points of this retrospective study. PFS was defined as the time from diagnosis to disease progression, relapse, or death from any cause, with censoring at the time of last follow‐up. OS was defined as the time from diagnosis to death from any cause, with censoring at the time of last follow‐up.

### 2.6 Statistical analysis

Receiver operating characteristics (ROC) analysis was used to determine the optimal cutoff of ΔSUVmax and ΔSUVmax*ΔSLD for survival prediction. Sensitivity, specificity, and accuracy were calculated for each iPET/CT interpretation method.

Estimates of survival were calculated using the Kaplan‐Meier method, and differences between groups were estimated using the log‐rank test. Univariate and multivariate Cox proportional hazards analyses were performed to examine the potential independent impacts of iPET/CT and clinical variables on PFS and OS.

All statistical analyses were performed using SPSS statistical software v 22.0 (IBM, Armonk, NY) and GraphPad Prism 6 (GraphPad Software, Inc, La Jolla, CA), and a *P* < 0.05 was considered statistically significant.

## 3 RESULTS

### 3.1 Patient characteristics

A total of 129 patients with PET/CT images acquired at baseline were screened. Only eight patients performed iPET/CT after two cycles of chemotherapy, and 77 patients obtained iPET/CT after four cycles of chemotherapy. Therefore, 77 patients with iPET4 were finally enrolled, and their clinical characteristics are presented in Table 1. After a median follow‐up of 23 months (range 4‐61 months), 27 patients progressed, and 22 patients died of progression.

**Table 1:** Patient clinical characteristics

Variable	Number of patients (%)
Age (range)	52 (22‐79)
Gender	
Male	43 (56)
Female	34 (44)
Ann Arbor stage	
I‐II	29 (38)
III‐IV	48 (62)
Lactate dehydrogenase (LDH)	
Normal	58 (75)
Abnormal	19 (25)
B symptoms	
Present	17 (22)
Absent	60 (78)
International prognostic index (IPI)	
Low risk (0‐1 factor)	33 (43)
Low‐intermediate risk (2 factors)	23 (30)
High‐intermediate risk (3 factors)	16 (21)
High risk (4‐5 factors)	5 (6)
Bone marrow involvement	
Present	16 (21)
Absent	61 (79)
Cell of origin subtypes	
GCB	33 (43)
Non‐GCB	42 (54)
Unknown	2 (3)
Double expression of MYC/BCL2	
Yes	13 (17)
No	13 (17)
Unknown	51 (66)

Abbreviation: GCB, germinal center B‐cell.

### 3.2 iPET/CT results

iPET/CT results according to different interpretation methods are presented in Table 2. As many previous studies have suggested, in DS visual analysis, iPET/CT with FDG uptake greater than that in the liver (score 4‐5) was positive, and those with uptake not greater than liver uptake (score 1‐3) were negative in the present study. Of a total of 77 patients, 31 (40%) had a positive iPET/CT, and 46 (60%) had a negative iPET/CT outcome (score 1‐3). Of the 27 patients who progressed, 16 (59%) had positive iPET/CT findings, and 11 (41%) had negative iPET/CT results.

**Table 2:** Correlation between visual and semiquantitative analysis for iPET/CT interpretation

	Deauville criteria		
	Negative, n (%)	Positive, n (%)	All, n (%)
ΔSUVmax (cutoff, 74%)			
Negative, n (%)	42 (55)	10 (13)	52 (68)
Positive, n (%)	4 (5)	21 (27)	25 (32)
All	46 (60)	31 (40)	77
ΔSUVmax*ΔSLD(cutoff, 30%)			
Negative, n (%)	39 (56)	11 (15)	50 (71)
Positive, n (%)	2 (3)	18 (26)	20 (29)
All	41 (59)	29 (41)	70

The optimal cutoff value of ΔSUVmax was 74% determined by ROC analysis, with an area under the raw ROC curve of 0.65 (*P* = 0.03). During the treatment, 25 patients (25/77, 32%) achieved a positive iPET/CT with ΔSUVmax < 74%, and 52 patients (52/77, 68%) achieved a negative iPET/CT with ΔSUVmax ≥ 74%. Of the 27 progressed patients, 16 patients (59%) had ΔSUVmax < 74%, and 11 (40%) had ΔSUVmax ≥ 74%. Among four patients with negative DS but positive ΔSUVmax on iPET/CT, three patients (75%) progressed during the follow‐up. In contrast, among the 10 patients with DS‐positive but ΔSUVmax‐negative iPET/CT findings, only three patients (30%) progressed during the follow‐up (Table 3).

**Table 3:** Outcome of subsets of patients defined by iPET/CT combing visual and semiquantitative analysis

I‐PET/CT		n	Treatment failure (progression or relapse)
Visual analysis	Semiquantitative analysis		
DS 1‐3	ΔSUVmax ≥ 74%	42	8 (19%)
DS 1‐3	ΔSUVmax < 74%	4	3 (75%)
DS 4‐5	ΔSUVmax ≥ 74%	10	3 (30%)
DS 4‐5	ΔSUVmax < 74%	21	13 (62%)
DS 1‐3	ΔSUVmax*ΔSLD ≥ 30%	39	10 (25%)
DS 1‐3	ΔSUVmax*ΔSLD < 30%	2	1 (50%)
DS 4‐5	ΔSUVmax*ΔSLD ≥ 30%	11	3 (27%)
DS 4‐5	ΔSUVmax*ΔSLD < 30%	18	12 (67%)

Abbreviation: DS, Deauville 5‐point scale.

The optimal cutoff of ΔSUVmax*ΔSLD was 30%, as determined by ROC analysis, with an area under the raw ROC curve of 0.63 (*P* = 0.07). Because the lesions of seven patients were unmeasurable (three due to bone involvement, four due to gastrointestinal involvement), 70 patients were finally evaluated by the ΔSUVmax*ΔSLD method. During treatment, 20 patients (20/70, 29%) achieved a positive iPET/CT result with ΔSUVmax*ΔSLD < 30%), and 50 patients (50/70, 71%) achieved a negative iPET/CT result with ΔSUVmax*ΔSLD ≥ 30%. Of the 26 progressed patients with measurable lesions, 13 patients (50%) had ΔSUVmax*ΔSLD < 30%, and 13 (50%) had ΔSUVmax*ΔSLD ≥ 30%. Of the two patients with negative DS but positive ΔSUVmax*ΔSLD iPET/CT results, one patient (50%) progressed during the follow‐up. In contrast, of the 11 patients with DS‐positive but ΔSUVmax*ΔSLD‐negative iPET/CT findings, only three patients (27%) progressed during the follow‐up (Table 3).

### 3.3 PFS and OS according to iPET/CT results

**Figure 1:**
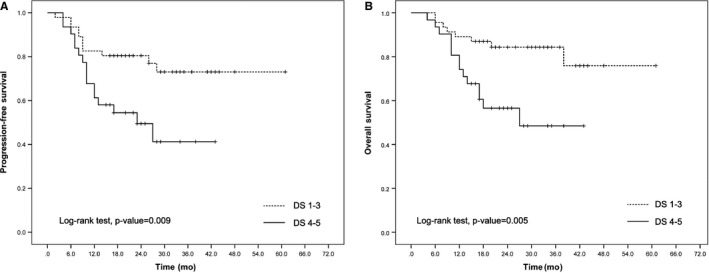
Kaplan‐Meier curves of progression‐free survival (A) and overall survival (B) of diffuse large B‐cell lymphoma patients according to Deauville 5‐point scale (DS)

**Figure 2:**
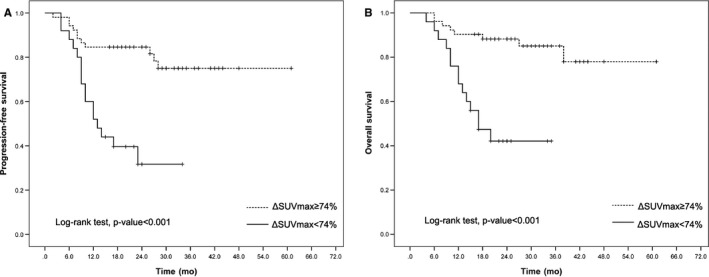
Kaplan‐Meier curves of progression‐free survival (A) and overall survival (B) of diffuse large B‐cell lymphoma patients according to ΔSUVmax (cutoff, 74%)

**Figure 3:**
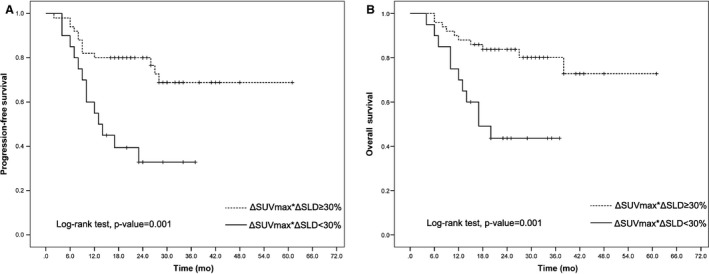
Kaplan‐Meier curves of progression‐free survival (A) and overall survival (B) of diffuse large B‐cell lymphoma patients according to ΔSUVmax*ΔSLD (cutoff, 30%)

After a median follow‐up of 23 months, the 2‐year PFS and OS were significantly different between the iPET/CT‐positive and iPET/CT‐negative patients regardless of iPET/CT interpretation by visual analysis or semiquantitative analyses (Figures 1, 2, 3).

According to the DS interpretation, the 2‐year PFS was 85% for patients with negative iPET/CT and 49% for patients with positive iPET/CT (*P* = 0.009; Figure 1A). The 2‐year OS was 84% for patients with negative iPET/CT and 56% for patients with positive iPET/CT (*P* = 0.005; Figure 1B).

The results for the semiquantitative analysis using ΔSUVmax were as follows: 2‐year PFS was 84% for patients with ΔSUVmax ≥ 74% in contrast to 31% for patients with ΔSUVmax < 74% (*P* < 0.001; Figure 2A); 2‐year OS was 88% for patients with ΔSUVmax ≥ 74% in contrast to 42% for patients with ΔSUVmax < 74% (*P* < 0.001; Figure 2B).

The semiquantitative analysis using ΔSUVmax*ΔSLD also strongly predicted 2‐year PFS and OS: 2‐year PFS was 80% for patients with ΔSUVmax*ΔSLD ≥ 30% in contrast to 33% for patients with ΔSUVmax*ΔSLD < 30% (*P* = 0.001; Figure 3A); the 2‐year OS was 84% for patients with ΔSUVmax*ΔSLD ≥ 30% in contrast to 44% for patients with ΔSUVmax*ΔSLD < 30% (*P* = 0.001; Figure 3B).

**Figure 4:**
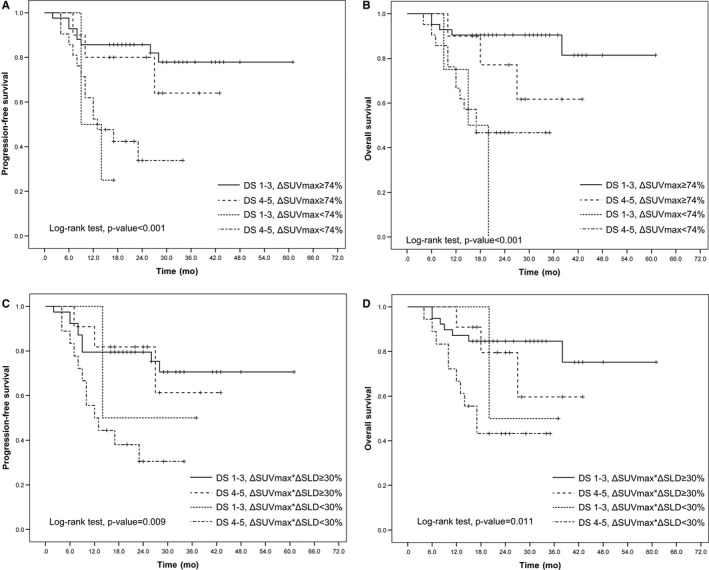
Progression‐free survival (PFS) and overall survival (OS) according to combination of visual and semiquantitative assessment. PFS (A) and OS (B) according to combination of DS and ΔSUVmax; PFS (C) and OS (D) according to combination of DS and ΔSUVmax*ΔSLD

Furthermore, the combined visual and semiquantitative analysis revealed that patients who remained DS‐positive and had a ΔSUVmax < 74% had a significantly poorer PFS (*P* < 0.001; Figure 4A) and OS (*P* < 0.001; Figure 4B) than patients who had a negative DS score or achieved a ΔSUVmax ≥ 74%. Patients who remained DS‐positive and had a ΔSUVmax*ΔSLD < 30% had a significantly poorer PFS (*P* = 0.009; Figure 4C) and OS (*P* = 0.011; Figure 4D) than did patients with a negative DS score or a ΔSUVmax*ΔSLD ≥ 30%.

The sensitivity, specificity, NPV, PPV, and accuracy for each criterion predicting disease progression and death are shown in Table 4. ΔSUVmax (cutoff, 74%) showed the best accuracy in predicting both PFS and OS.

**Table 4:** Sensitivity and specificity of three criteria

	Deauville 5‐point scale (%)	ΔSUVmax (cutoff, 74%) (%)	ΔSUVmax*ΔSLD (cutoff, 30%) (%)
PFS^a^			
Sensitivity	59	59	50
Specificity	70	82	84
PPV	52	64	65
NPV	76	79	74
Accuracy	66	74	71
OS^b^			
Sensitivity	45	64	52
Specificity	83	80	82
PPV	64	56	55
NPV	69	85	80
Accuracy	68	75	73

Abbreviations: NPV, negative predictive value; PPV, positive predictive value.

a Progression or relapse events n = 27.

b Deaths n = 22.

### 3.4 Univariate and multivariate analyses for predictors of survival

**Figure 5:**
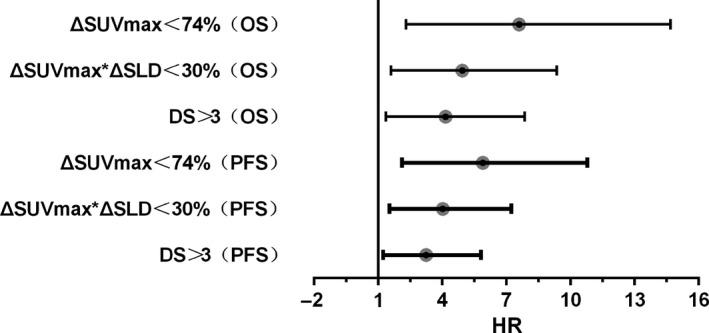
Comparison of hazard ratios (high‐ versus low‐risk groups) and 95% confidence intervals for various iPET/CT interpretation methods, including Deauville 5‐point scale (DS), ΔSUVmax, and ΔSUVmax*ΔSLD

**Figure 6:**
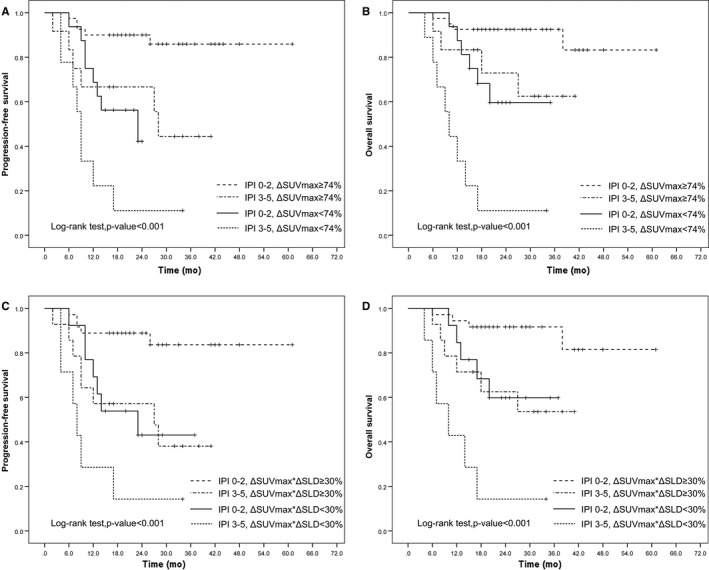
Progression‐free survival (PFS) and overall survival (OS) according to combination of IPI score and ΔSUVmax‐based method. PFS (A) and OS (B) according to combination of IPI and ΔSUVmax; PFS (C) and OS according to combination of IPI and ΔSUVmax*ΔSLD

Univariate and multivariate analyses were performed to identify predictors of survival outcomes (Tables 5 and 6). The univariate analyses showed that the following variables were statistically significant: IPI, DS, ΔSUVmax*ΔSLD, and ΔSUVmax. The iPET/CT results based on all three interpretation methods exhibited prognostic significance for PFS and OS (Figure 5). However, the prognostic significance of DS and ΔSUVmax*ΔSLD was diminished in the multivariate analysis. In the multivariate analysis, IPI and ΔSUVmax remained independent predictors of PFS and OS. The PFS and OS curves of patients grouped according to the results of ΔSUVmax‐based methods in combination with IPI score are shown in Figure 6. These results illustrated that the worst patient outcomes belonged to the iPET/CT‐positive and IPI high‐risk groups.

**Table 5:** Univariate analysis of prognostic factors for PFS and OS

	PFS			OS		
	HR	95% CI	*P* value	HR	95% CI	*P* value
Gender (male vs female)	1.55	0.73‐3.29	0.258	1.24	0.53‐2.87	0.619
IPI score (≥3 vs 0‐2)	4.34	1.87‐10.78	<0.001*	4.34	1.87‐10.08	0.001*
Bone marrow involvement (yes vs no)	1.07	0.43‐2.66	0.880	1.43	0.56‐3.65	0.460
Deauville 5‐point scale (score 4‐5 vs score 1‐3)	2.68	1.23‐5.81	0.013*	3.26	1.36‐7.85	0.008*
ΔSUVmax (<74% vs ≥74%)	4.78	2.12‐10.79	<0.001*	5.81	2.29‐14.68	<0.001*
ΔSUVmax*ΔSLD (<30% vs ≥30%)	3.32	1.53‐7.23	0.002*	3.86	1.59‐9.36	0.003*

Abbreviations: CI, confidence interval; HR, hazard ratio; IPI, International prognostic index; OS, overall survival; PFS, progression‐free survival.

* *P* < 0.05.

**Table 6:** Multivariate analysis of prognostic factors for PFS and OS

	PFS			OS		
	HR	95% CI	*P* value	HR	95% CI	*P* value
IPI score (≥3 vs 0‐2)	3.34	1.49‐7.45	0.003*	4.53	1.84‐11.15	0.001*
Deauville 5‐point scale (score 4‐5 vs score 1‐3)	0.55	0.17‐1.73	0.305	0.54	0.15‐1.89	0.332
ΔSUVmax (<74% vs ≥74%)	5.26	1.47‐19.06	0.011*	9.77	2.25‐42.36	0.002*
ΔSUVmax*ΔSLD (<30% vs ≥30%)	1.41	0.42‐4.74	0.575	1.05	0.28‐3.87	0.946

Abbreviations: CI, confidence interval; HR, hazard ratio; IPI, International prognostic index; OS, overall survival; PFS, progression‐free survival.

* *P* < 0.05.

## 4 DISCUSSION

In this study, we attempted to evaluate the prognostic value of iPET/CT according to visual and semiquantitative interpretation methods in newly diagnosed DLBCL patients treated with CHOP ± R. The results show that patients with positive iPET/CT had a significantly poorer outcomes than those of patients who achieved negative iPET/CT both in terms of PFS and OS, irrespective of the iPET/CT interpretation method (DS, ΔSUVmax, or ΔSUVmax*ΔSLD). The semiquantitative approach based on ΔSUVmax is more accurate than visual analysis for identifying patients with different prognoses.

Although ^18^F‐FDG‐PET has been widely used for staging and determining the final response of DLBCL, the role of iPET/CT is still controversial, which is mainly attributable to a lack of standardized interpretation methods.^24^ DS is intended as a simple, reproducible scoring method and is recommended for reporting PET/CT.^4^ However, more recent studies have demonstrated that iPET/CT assessed by DS shows excellent negative predictive value (NPV) but variable PPV.^9^, ^10^, ^11^ As reported by Moskowitz et al, biopsy of iPET/CT‐positive residual masses revealed a very high false PPV in 87% of cases, and iPET/CT failed to predict the outcomes.^25^ In contrast, some other studies illustrated that iPET/CT with a relatively high PPV had the ability to identify high‐risk patients and effectively predict outcomes.^6^, ^26^ In our study, PPV generated by the DS was 51.61%, and iPET/CT evaluated by DS showed a significant prognostic value of 2‐year PFS and OS. It seems that the low PPV of visual analysis weakens the predictive value of iPET/CT. The drop in PPV may be related to improved outcomes with rituximab or novel agents for modern management of DLBCL.^10^, ^11^, ^25^, ^27^, ^28^ A different measurement or combination of factors may be required to improve visual analysis.

Increasing evidence has illustrated that the semiquantitative method using SUVmax reduction is more accurate than visual analysis, based on DS to identify subgroups of patients with significantly different prognoses.^6^, ^14^, ^15^, ^16^, ^17^ Our results are consistent with those of other studies and confirm that ΔSUVmax evaluation (cutoff value of 74%) of iPET/CT is an independent predictive factor for DLBCL patients. Survival analysis revealed that patients with ΔSUVmax ≥ 74% had a significantly better PFS and OS than did patients with ΔSUVmax < 74%, irrespective of DS scores (Figure 4). Combining ΔSUVmax and DS assessment helps to further identify false‐positive results assessed by DS alone. The cutoff value of previously reported is variable. In the study of Casasnovas et al, the cutoff was 66% for PET0‐2 and 70% for PET0‐4.^13^ Rossi et al reported a cutoff value of 71% for PET0‐2.^29^ The similarity of the ΔSUVmax cutoff value in our study and previous studies suggests that these thresholds appear to be robust and reproducible regardless of DLBCL patients treated with either the CHOP or CHOP‐like regimen combined with or without rituximab.^13^ Although ΔSUVmax analysis improves PPV, false‐positive results can also be generated to a lesser extent. This could be attributed to a low baseline SUVmax (<10.0) leading to SUVmax reduction of less than the cutoff value.^14^ In the present study, eight patients had a ΔSUVmax <74% but no PD, and six (75%, 6/8) had a baseline SUVmax less than 10.

Tumor burden is an important prognostic factor in malignant lymphoma patients.^18^ Quantitative imaging methods using PET/CT to evaluate tumor burden are under development. Parameters including MTV, total lesion glycolysis (TLG), ΔMTV, and TLG reduction (ΔTLG) have shown promising results in predicting outcomes for DLBCL patients.^18^, ^30^, ^31^ However, some limitations exist in the measurement of MTV: special software used to segment tumors is needed; there is no consensus on the optimal measurement methods; and the process is time‐consuming and cannot be performed in routine daily practice.^32^ In Hodgkin lymphoma, the combination of changes in node size on CT and SUVmax on PET has been explored and may predict disease progression and relapse.^19^, ^20^ Investigators examined the size‐incorporated maximum standardized uptake value (SIMaxSUV), defined as the product of SUVmax and the greatest lesion diameter, and found that a high SIMaxSUV is the single most important predictor for disease‐free survival and overall survival in DLBCL patients.^33^ In the present study, ΔSUVmax*ΔSLD is defined as the change in the product of ΔSLD and ΔSUVmax, which is similar to the change in SIMaxSUV. ΔSLD is easily measured and reproducible. Accordingly, iPET/CT evaluated using ΔSUVmax*ΔSLD can identify significantly different prognostic groups. ΔSUVmax*ΔSLD less than 30% predicts poor PFS and OS in the present population. Regrettably, ΔSUVmax*ΔSLD failed to be independently predictive for PFS and OS when the multivariate analysis was performed. Thus, the value of ΔSUVmax*ΔSLD interpretation of iPET/CT data needs to be further explored, and more prospective trials are warranted.

Using iPET/CT as a prognostic tool should be complementary to the existing procedures, especially IPI.^6^ Although the prognostic accuracy of IPI has been influenced by the results of new treatment modalities, such as CD20 monoclonal antibodies, IPI is still recommended and widely used as a standard for predicting different outcomes in DLBCL patients.^34^ In the present study, we confirmed that the IPI and iPET/CT interpreted using the ΔSUVmax method are independent prognostic factors. Among the patients with high‐risk IPI, the patients with an iPET/CT‐positive response had an extremely poor prognosis compared to those with an iPET/CT‐negative response for OS and PFS. The results are consistent with those of previous studies that reported that patients with ΔSUVmax lower than the cutoff value had poor OS and PFS, and iPET/CT based on ΔSUVmax analysis was an independent prognostic factor for DLBCL patients.^13^, ^14^

The present study has some limitations of note. First, this is a single‐center retrospective study. Second, the sample size is not very large. Third, the treatment regimens varied among our cohort of patients. The results may thus have been influenced by the heterogeneous population and varied treatment. The predictive value of iPET/CT is limited in this study, and larger prospective trials are needed.

In conclusion, our encouraging results suggest that the use of semiquantitative analysis in addition to visual analysis may aid in the interpretation of interim PET/CT findings for DLBCL patients undergoing CHOP ± R as a first‐line treatment. The ΔSUVmax analysis shows the best accuracy and the strongest predictive value to identify patients with different prognoses among these three methods. ΔSUVmax*ΔSLD, a parameter that combines changes in SUVmax and tumor size, may be a promising method to interpret iPET/CT findings. Further randomized, large prospective studies are warranted to confirm these results.

## CONFLICTS OF INTEREST

None declared.

## Data Availability

The data used to support the findings of this study are available from the corresponding author upon request.
